# Revisiting Persistent Neuronal Activity During Covert Spatial Attention

**DOI:** 10.3389/fncir.2021.679796

**Published:** 2021-06-30

**Authors:** Julian L. Amengual, Suliann Ben Hamed

**Affiliations:** Institut des Sciences Cognitives Marc Jeannerod, CNRS UMR 5229, Université Claude Bernard Lyon I, 67 Boulevard Pinel, Bron, France

**Keywords:** spatial attention, prefrontal cortex, mixed-selectivity, population activity, decoding, neurophysiology, persistent activity, alpha oscillations

## Abstract

Persistent activity has been observed in the prefrontal cortex (PFC), in particular during the delay periods of visual attention tasks. Classical approaches based on the average activity over multiple trials have revealed that such an activity encodes the information about the attentional instruction provided in such tasks. However, single-trial approaches have shown that activity in this area is rather sparse than persistent and highly heterogeneous not only within the trials but also between the different trials. Thus, this observation raised the question of how persistent the actually persistent attention-related prefrontal activity is and how it contributes to spatial attention. In this paper, we review recent evidence of precisely deconstructing the persistence of the neural activity in the PFC in the context of attention orienting. The inclusion of machine-learning methods for decoding the information reveals that attention orienting is a highly dynamic process, possessing intrinsic oscillatory dynamics working at multiple timescales spanning from milliseconds to minutes. Dimensionality reduction methods further show that this persistent activity dynamically incorporates multiple sources of information. This novel framework reflects a high complexity in the neural representation of the attention-related information in the PFC, and how its computational organization predicts behavior.

## Introduction

Numerous studies report an increase of spiking activity in different brain areas during the performance of visual delayed tasks [see Fuster and Alexander (1971), Goldman-Rakic (1995), Shafi et al. (2007), Barak et al. (2010), Watanabe and Funahashi (2014), Chaudhuri and Fiete (2016), Zylberberg and Strowbridge (2017), Manohar et al. (2019), for a review]. The general structure of the tasks consists of the presentation of an informative visual cue about how the subject should act afterward. After the presentation, there is a delay period in which the subject must keep in mind the information provided by the cue to appropriately respond to the task demands. This information can be spatial (e.g., left vs. right), feature-based (e.g., blue vs. red), or symbolic (e.g., left-pointing arrow vs. right-pointing arrow).

Pioneering electrophysiological studies employing intracortical recordings in non-human primates have identified neurons that not only show activity associated with the sensory stimuli serving as a cue but also show activity in the delay period after the cue when it is no longer present and the task instructions are being processed (Fuster and Alexander, 1971; Fuster, 1973; Funahashi et al., 1989; Miller et al., 1993). These findings have been extensively corroborated by using different protocols and techniques in humans and non-human primates (Constantinidis et al., 2018 for review). Classically, persistent activity in the prefrontal cortex (PFC) has been considered as a signature of specific cognitive processes such as working memory (Constantinidis et al., 2018). However, working memory interplays with other cognitive functions such as perception or attention. For example, previous studies have succeeded in discriminating perceptual and mnemonic representations of visual features (Mendoza-Halliday and Martinez-Trujillo, 2017a). How the interaction between working memory and attention is theorized depends on whether attention is conceptualized as the processing of a limited source of information (a perception of low-salience visual information) or the selection of information for processing [covert attention: Oberauer (2019)]. In the present review, we will focus on covert attentional processes defined as the *a priori* top-down selection and maintenance of the sensory information for prioritization (e.g., based on its spatial location), in anticipation of its presentation and processing. In this context, covert attention can be considered as an instance of working memory as the information needed to be prioritized, whether feature-based or spatial, is by definition sustained, i.e., held in working memory (Desimone and Duncan, 1995). We will describe the structure and informational content of the observed neuronal activity in the deployment of covert attention, and discuss it in relation to the current views on the dynamic and rhythmic nature of attention [see Gaillard et al. (2020), Gaillard and Ben Hamed (2020) for a review].

**Figure 1:**
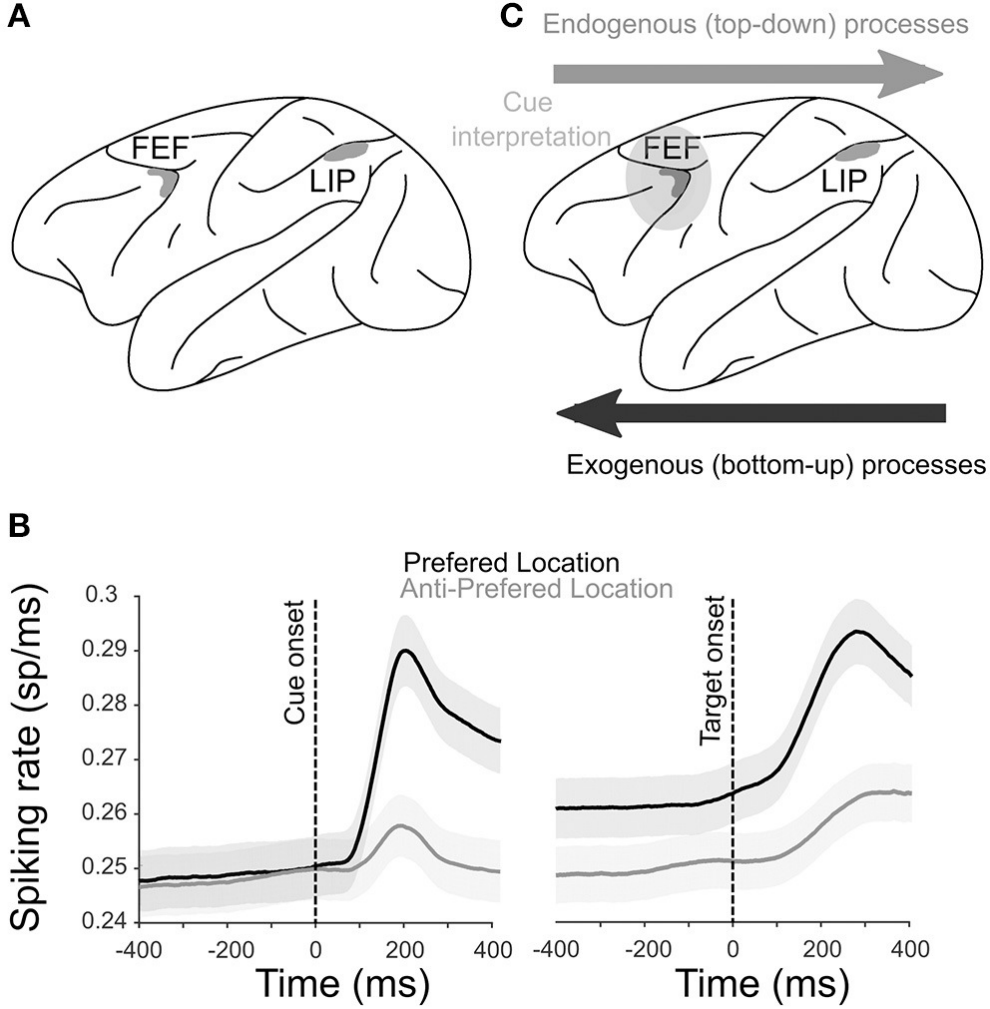
Physiology of the attentional system. **(A)** Anatomical localization of the two core brain regions engaged during spatial attention orienting in the macaque [shaded in gray; see Cohen and Andersen (2002), Ibos et al. (2013), Paneri and Gregoriou (2017), for a review], the FEF and the LIP sulcus. **(B)** The average multiunit activity (MUA; ± s.e.) recorded from the FEF in one monkey when a cue is orienting attention toward the preferred (black) or the anti-preferred (gray) spatial location locked to cue (left) and target (right) onsets. *X*-axis represents the time around cue or target onsets. **(C)** Functional hierarchy in a frontoparietal network during attentional processes. Exogenous processes start in the LIP and project to the FEF. Cue interpretation takes place in the FEF, and the selection of the spatial location and subsequent endogenous processing is projected from the FEF to the LIP.

Persistent activity during visuospatial attention tasks has been reported both in parietal (Colby et al., 1996; Gottlieb et al., 1998; Ibos et al., 2013) and prefrontal cortices (Moore and Armstrong, 2003; Moore and Fallah, 2004). The tasks involve maintaining a sustained level of information relative to where (spatial attention) or what (feature-based attention) relevant task-related events will need to be processed (Posner and Petersen, 1990). Attention orienting can be driven by bottom-up or stimulus-driven processes, triggered by the salience of the incoming visual stimuli (i.e., their shape or color), and a top-down process that is guided by the relevance of the stimulus (i.e., how much it is useful to the task) defining our internal goals or expectations (Pinto et al., 2013; Katsuki and Constantinidis, 2014). Studies on humans have highlighted the importance of a frontoparietal network in the control of attention, showing the involvement of the parietal cortex and the PFC (Corbetta and Shulman, 2002). In macaque monkeys, the most commonly used model to study the attentional system in non-human primates, a homologous frontoparietal attention network is identified (Figure 1A), involving the lateral intraparietal (LIP) area (Gottlieb et al., 1998) and the frontal eye field (FEF; Armstrong et al., 2009; Monosov and Thompson, 2009). The two cortical regions are highly interconnected (Cavada and Goldman-Rakic, 1989; Stanton et al., 1995; Buschman and Miller, 2007; de Schotten et al., 2011; Ibos et al., 2013; Marek, 2018). Reversible inactivation of the two cortical regions results in behavioral impairments both in easy visual search tasks that rely on bottom-up attentional processes (Wardak et al., 2002; Wardak, 2006) and in conjunction with the search tasks that involve top-down attentional processes (Theeuwes, 1993). Although persistent activity has also been described in other regions, including the regions where it is more prevalent compared to the FEF and LIP (Leavitt et al., 2017), we focus on neuronal activity in these two regions in the context of persistent activity during attention orienting.

## Persistent Neuronal Activity During Spatial Attention Orienting

Persistent activity during sustained attentional processes is classically described in both the FEF and LIP. Figure 1B shows the average neuronal responses of a sample of FEF attention-related neurons recorded during the cue-to-target interval of a spatial attention task. A higher activation is observed when the cue is orienting attention toward the preferred spatial position of the neuron (black) compared to when attention is oriented away from the preferred spatial position (gray). Preferred spatial positions coincide with both enhanced visual cue-related responses as well as enhanced visual target detection responses. Such neuronal response patterns are typical of both FEF and LIP neurons (Ibos et al., 2013). As a result, an important question in the field has been to understand whether parietal and prefrontal attentional responses were functionally identical or not. Simultaneous recordings from both cortical regions allow addressing this question. In the following sections, we will first review this question from the point of view of a single neuron persistent activity and then from the perspective of the neuronal population.

### Attention-Related Persistent Responses in Single Neurons

Several studies have addressed the functional interactions between the PFC and the parietal cortex during attentional processes. In easy visual search tasks (e.g., detecting a red square among the green squares), which have been shown to rely on bottom-up attentional processes (Treisman and Gelade, 1980), parietal neurons are activated earlier than prefrontal neurons (Buschman and Miller, 2007). In striking contrast, in conjunction with the visual search tasks (e.g., detecting an orange vertical bar among the red vertical bars and red and orange horizontal bars), which have been shown to rely on top-down attentional processes (Treisman and Gelade, 1980), the reverse is observed (Buschman and Miller, 2007). This suggests that spatial attention or spatial selection mechanisms flow from the parietal cortex to the PFC and the PFC to the parietal cortex when driven by the environment and the subject's internal goals, respectively. However, visual search tasks do not allow researchers to dissociate the neuronal processes related to attention orientation from those related to perceptual cue processing. In order to address this limitation, Ibos et al. (2013) designed a task that allows to temporally dissociate between cue processing, cue interpretation and attention orientation, and target selection. This task had two features (Supplementary Figure 1). It was based on a modified version of a rapid serial visual presentation (RSVP) task (Potter, 2018) such that, on each trial, the cue and the target are embedded in two parallel continuous streams (succession) of isoluminent distractors. In such a context, both parietal and prefrontal neurons do not respond to the visual transients between one visual stimulus and the next. Thus, any specific enhancement of neuronal responses to the cue or to the target or in between the cue and the target can be interpreted as an attention orientation signal or a perceptual signal. The second specificity of this task lies in the fact that the attentional orientation cues are highly symbolic. The green cues indicate that the target will appear in the same visual stream as the normal cue while the red cues indicate that the target will appear in the opposite visual stream. In other words, both the left red cues and right green cues oriented attention to the right while both the right red cues and left green cues oriented attention to the left. While both parietal and prefrontal neurons showed an enhanced processing of the cues and targets embedded in the RSVP streams, the cue-related responses had shorter latencies in the parietal cortex than in the PFC, and the target-related responses had shorter latencies in the PFC than in the parietal cortex. Thus, this confirms the idea that spatial selection mechanisms flow from the parietal to the PFC and the PFC to the parietal cortex when driven by the environment and by the subject's internal goals, respectively (Figure 1C). In addition, neurons explicitly encoding the instruction for the spatial attention orientation independently of the color and location of a cue were only identified in the PFC and had longer response latencies than the cue-related parietal responses, indicating that the attentional cue interpretation was performed within the PFC (Figure 1C). Overall, this thus defined a clear hierarchical functional organization within the parietofrontal network in which the processing of high-saliency stimuli initiates in the LIP; and the active attention orientation control according to the subject's goals takes place in the FEF, thus driving a perception of low-saliency stimuli.

As shown by Ibos et al. (2013) and with relevance to the present review, the prefrontal attention orientation neurons encoded the attention instruction in a sustained manner. This was also the case of a substantial proportion of the cue-related neurons of both cortical regions that responded to one specific category of cues such as cue color or cue position. Thus, these neurons are also expected to contribute to the coding of attention orientation instructions when combined across the population. The fact that FEF neurons explicitly encode the cue instruction suggests functional differences of how both the FEF and LIP represent a spatial orientation signal in the population level and sustain these representations in time. It has been hypothesized that the ability of individual neurons in a recurrent neuronal network to sustain the information over time depends on the correlated fluctuations of activity within the local neuronal microcircuitry (Maimon and Assad, 2009). The recurrent fluctuations of neuronal activity occur over a wide range of timescales depending on the local properties of the brain region (Murray et al., 2014). To measure the timescales of these fluctuations, the time lag autocorrelogram of the spike count of individual neurons is calculated. As this time lag increases, the autocorrelation decays as a function of the fluctuation timescales (Churchland et al., 2011). The timescale of these fluctuations is mathematically characterized by the decay of autocorrelation as a function of the time lag (τ), which corresponds to the fitting of the autocorrelogram with an exponential decay and an offset. The intrinsic timescales differ across the brain areas, showing shorter timescale values in the parietal cortex and longer timescale values in the PFC (Murray et al., 2014). The observation points in the direction of favoring a temporal hierarchical organization between the parietal and the PFC (Murray et al., 2014). One question is whether and how this impacts the functional coding of the neuronal populations as a whole. This is explored in the next section.

### Population Activity

Single-neuron responses support an idea of the sustained/persistent neuronal activity (quantified as the sustained average spiking rate in time when computed across the trials) during delay epochs in cognitive tasks. What is the nature and origin of this “persistence” of persistent activity? Classical models of persistent activity propose that the observed spiking activity of a given neuron during the delay periods of a task is a marker of an active state of the neural ensemble it belongs to, which keeps the neural population available for processing the encoded information (Lundqvist et al., 2018a for a review). However, a closer inspection of the single neuron persistent activity reveals that a spiking activity is better characterized by sparsity than by persistence, suggesting that the information might be held within the local functional network by the changes in synaptic weights rather than in the spiking activity *per se* (Zucker and Regehr, 2002; Lundqvist et al., 2016). It is proposed that this type of information encoding during the delay period might be more long-lasting and more resistant to the disruption by additional inputs compared to a purely persistent spiking activity (Lundqvist et al., 2018b). This observation has been supported by the computational models that predict the sustainability of persistent activity through virtue of recurrent connections between the neurons that have an affinity for shared specific stimulus properties (Compte, 2000; Compte et al., 2003). However, it must be acknowledged that the absence of persistent activity can also be accounted for by analytical and experimental biases. For instance, one must take into account that each cortical neuron receives inputs from several other neurons (up to several thousands), which might cause a high response variability at small timescales but less so at longer timescales. Corroborating this view, Leavitt et al. (2017) show evidence of persistent activity during a working memory task using the temporal scales larger than 400 ms whereas the smaller timescales did not show such an effect. Another possible reason for the absence of persistent activity is the use of single-cell approaches, which, during the mapping of a given cortical area, might miss a specific region in which the persistent activity takes place. In this respect, it is, however, worth noting that sparsity in the spiking activity has been observed in the studies by using dense multielectrode recordings.

Thus, one of the implications of the above framework is that persistent activity is best understood in the level of the neuronal population rather than in the level of individual neurons. This view assumes, among other things, that information coding cannot be unambiguously read out (i.e., decoded) from the spiking rate of a single neuron, but is best characterized by the patterns of activation and connectivity across a population of neurons. That is to say, quoting from Averbeck et al. (2006), “*As in any good democracy, individual neurons count for little; it is population activity that matters.”* Accordingly, there is increasing evidence that individual neuronal response profiles do not fully mirror the dynamics of the functional neuronal population they belong to and that the dynamics of the connection weights between the individual neurons must be taken into account (Barak et al., 2010; Crowe et al., 2010; Stokes et al., 2013).

**Figure 2:**
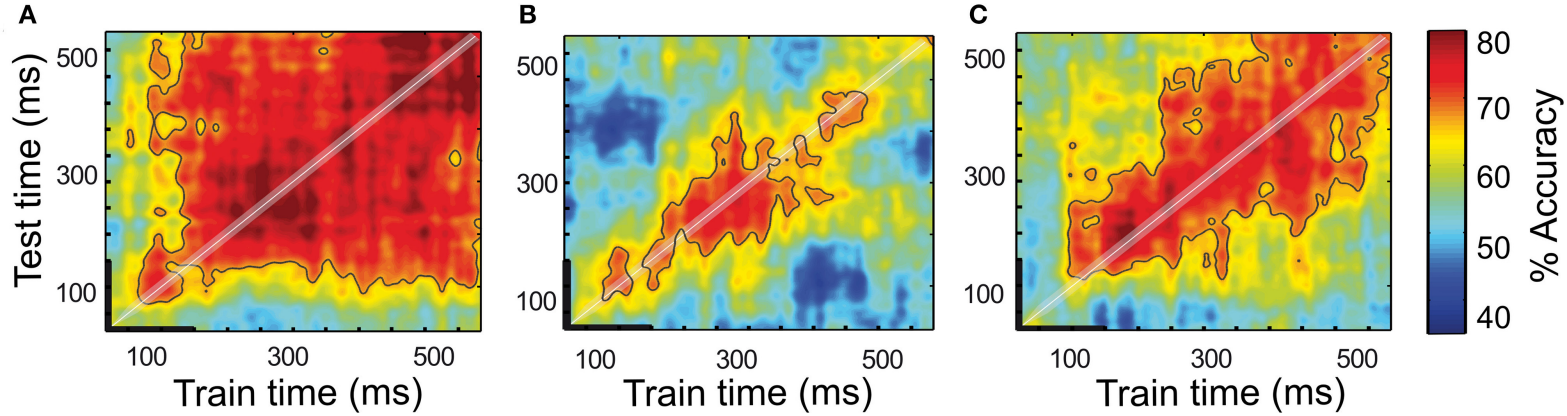
Temporal dynamics of a spatial attention signal. Full cross-temporal classification analysis of the attention-specific FEF population **(A)**, entire LIP population **(B)**, and entire FEF population **(C)**. Classifiers are trained to classify spatial attention from the population activities at every time step within 600 ms following a cue onset and prior to a target onset (*x*-axis, thick black line corresponds to the cue presentation). The performance of each classifier is tested from the population activities obtained from naïve trials at every time step within the same time period relative to the cue. Red colors represent high performance whereas the blue-colored regions represent low performance [chance level at 50%, figure adapted from Astrand et al. (2015)].

When using the cued target detection tasks to orient attention, it is often assumed that attention is behaviorally allocated in a stable and sustainable manner in the cued location. Likewise, it is assumed that the neurons that encode this information do so in a stable manner, i.e., with a constant number of spikes in time. However, the evidence points that neither of these assumptions might be correct (see the next section). One way to assess code stability in time is to use cross-temporal decoding approaches (King and Dehaene, 2014; Astrand et al., 2015; Varoquaux et al., 2017). These approaches assume that the attention orientation code is implemented by the neuronal population locked to the cue presentation. Thus, a decoder is trained at identifying whether attention is oriented toward one among multiple spatial locations (e.g., left vs. right) based on the neuronal population activities collected in a given (typically short, ~100 ms) time interval at a fixed delay from the cue presentation. The decoder is then tested at decoding attention orientation on the novel activities sampled all throughout the cue-to-target interval. If the decoder maintains a high decoding performance at all times (Figure 2A), then this indicates a stable code. Alternatively, if the decoder only achieves maximal decoding from the neuronal activities sampled at the same delay from the cue as the training activities, then this indicates a dynamic recurrent coding of attention orientation (Figure 2B). This means that the same cascade of neuronal activities unfolds throughout the cue-to-target activity from one trial to the next, reliably encoding spatial attention at each time but with a different neuronal code. Both a stationary regime and a dynamic regime can coexist in a given neuronal population, leading to a mixed cross-temporal decoding map (Figure 2C). Using a regularized linear regression classifier as a decoder (Astrand et al., 2014a), training on 70% of the available trials and testing it on the remaining 30% over multiple random draw repetitions (Ben Hamed et al., 2003), Astrand et al. (2015) show that, in the PFC, the neuronal population composed of the attention orientation cells represents the spatial attention orientation in a sustainable manner (Figure 2A). The entire task-related FEF neuronal population expresses a mixed cross-temporal decoding map, suggesting the combination of both stationary and dynamic processes (Figure 2C). Thus, this indicates that the functional characterization of the individual neuronal responses does not fully account for how the information is encoded in a given area. In contrast, the parietal neuronal population expresses a highly dynamic coding of the spatial attention orientation (Figure 2B). In other words, a parietal code for the spatial attention orientation changes from one time to the next.

Additionally, in the PFC, the mixed attention orientation coding coexists with a stationary code for a cue position and a highly dynamic code for a cue color. This indicates that a given functional neuronal population can concurrently code different sources of information in multiple coding regimes, respectively. In other words, the information is multiplexed, and the system is able to simultaneously process (multiplex) the different driving inputs that involve different neuromodulatory sensitivities and synchronization influences (Liu and Hou, 2013; Feng et al., 2014). At this point, it is important to disambiguate multiplexing of information from coding generalization. Whereas, the first refers to the ability of the neural population to simultaneously encode the different sources of information in multiple coding regimes, the second refers to the ability to decode the different sources of information by using the same code. Prior studies have shown evidence that the PFC neural population codes associated with one specific source of information do not fully generalize (Tremblay et al., 2015b; Mendoza-Halliday and Martinez-Trujillo, 2017b).

In a given cortical region, the specific neuronal coding regime (stationary vs. dynamic) might fully depend on the information to be encoded (e.g., attention orientation would always be encoded in a stationary manner while the color in a dynamic manner), as an intrinsic property of the neuronal population. Alternatively, this could actually be task-dependent (e.g., attention orientation would be encoded in a stationary manner in the cued target detection task, but dynamically in a spontaneous visual exploration task). This remains to be tested. Likewise, how these cortical areas read out these multiple codes and exploit them is a topic of future research. Multiple mechanisms might be at play. For example, similar to the previous working memory studies (Fujisawa et al., 2008; Mongillo et al., 2008), Astrand et al. (2015) propose that the active mechanisms that sustain the attentional information in the neural population might involve short-term plasticity mechanisms. In contrast, the constant inputs to the neuronal population might result in time-dependent response patterns if the synaptic weights that represent the connectivity across the neurons are continuously being changed by the influence of the input pattern of activity (Buonomano and Maass, 2009).

In this section, we have shown that whereas individual neurons in the PFC show a persistent average activity and the underlying neural population encodes the information using different regimes spanning from fully stationary to dynamic and mixed. This calls for a reinterpretation of the persistent activity at a single-neuron level during spatial attention orienting. In the next section, we show that, at the single-trial level, neither the single-neuron responses nor the neuronal population information is persistent.

## Is Persistent Attention-Related Information Actually Persistent?

### Single Trial, Spatially, and Temporally Resolved Access to Attention Selection Signals

The use of classification procedures to decode the brain activity associated with specific aspects of human behavior forms the basis of one of the greatest technological achievements in neuroscience for the last two decades, namely brain–computer interfaces (BCIs) (Chapin et al., 1999; Wolpaw et al., 2002). These methods are based on the use of simultaneous neuronal population activities from a given cortical region in order to drive the devices that can help patients with specific dysfunctions or deficits to improve their quality of life. Most BCI technologies are designed to address motor-related dysfunctions such as motor prosthesis or driving external palliative devices such as cursors (Trejo et al., 2006) or robotic arms (Sunny et al., 2016), among others. Little research has been directed to develop the BCI devices that rely on decoding higher-order cognitive processes such as attention (Andersen et al., 2010; Astrand et al., 2014b), due to the fact that such a cognitive content is internally generated by the neuronal signals that are often multiplexed with different types of information, including sensory and motor information. This renders their real-time access very challenging.

**Figure 3:**
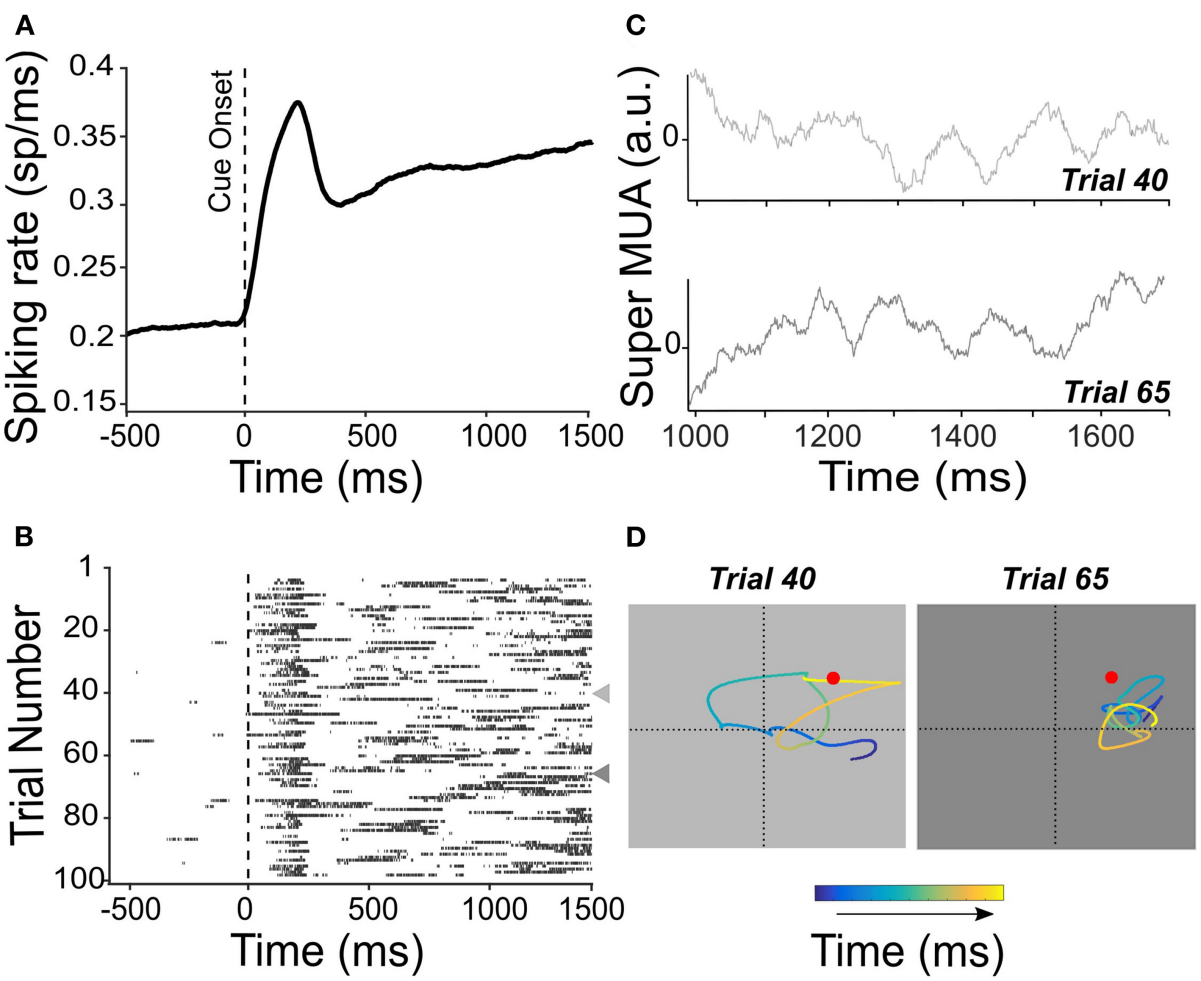
Persistent activity is not persistent from one trial to the next. **(A)** Mean spiking rate activity across 100 trials is recorded from the FEF. Activity is locked to the cue presentation in the preferred location of the neuron. The spiking rate shows an average and sustained increase during the cue-to-target interval. **(B)** Raster plot showing the multiunit activity (MUA) locked to the cue onset for each of the 100 trials used for the average spiking rate shown in **(A)**. Represented individual trial spiking probability sets at a threshold of 65%. Trials 40 and 65 are indicated by a leftward-pointing gray triangle to show a different pattern of temporal activation. These trials are also considered in **(C,D)**. **(C)** The average MUA activity was obtained by averaging the activity recorded from all channels of the same electrode [super MUA, left FEF see Gaillard et al. (2020)] for trials 40 and 65 [indicated in **(B)**]. **(D)** Attentional traces were obtained from high-resolution *x*-, *y*-coordinate decoding of the position of the attentional spotlight from the population neuronal activity, for trials 40 and 65, during the cue-to-target interval. Color code shows the time point along the cue-to-target interval of the decoded position of attention (blue to yellow, 700 ms). Red dot indicates the position of the target stimulus in these trials.

Non-human primate studies addressing this question have specifically targeted the cortical regions in which spatial attention has been shown to be sustained, thus favoring the PFC over the parietal cortex. Astrand et al. (2014a) first demonstrated a single-trial left/right attention classification for comparing multiple classifiers. Tremblay et al. (2015a) extended these observations to a four-quadrant classification of attention. Astrand et al. (2016) push this decoding procedure one major step forward, introducing the highly spatially resolved (*x, y*) tracking of the attentional spotlight [i.e., the actual portion of space being selected (Posner and Petersen, 1990)], at a spatial resolution of the order of 0.1° (see Supplementary Figure 2 for a description of the methods). Specifically, a regularized optimal linear estimator is used to associate the recorded bilateral response patterns produced during the correct target detection trials shortly before a target onset with the cued two-dimensional (*x, y*) spatial location of attention. This decoder is used to predict the (*x, y*) location of the attentional position inferred from the bilateral prefrontal response patterns recorded in novel trials naïve to the decoder. Decoding is applied at multiple time steps, thus allowing to track the attentional spotlight in time during the cue-to-target period. Using this methodological approach, attention could be decoded everywhere on the workspace during the cue-to-target presentation interval, and not necessarily static at the cued location prior to the target presentation (Astrand et al., 2016, 2020). This corresponds to the distinct neuronal response patterns on successive trials. Therefore, although the average neuronal response patterns might seem to be sustained (Figure 3A), on individual trials, spiking probability varies from one trial to the next (Figure 3B), corresponding to a different attentional exploration trace from one trial to the next (Figures 3C,D).

Importantly, Astrand et al. (2016) show that attention had a higher probability of being closer to the target on the correct target detection trials than on the trials in which the target is missed. Likewise, attention had a higher probability of being closer to a distractor when a false response to the distractor (as opposed to being closer to the target) was performed. This observation was further confirmed on other tasks (Di Bello et al., 2020; Gaillard et al., 2020). In this context, De Sousa et al. (2021) further enhance a correlation between the decoded attention and overt behavior using a novel two-step decoder, the essence of which is to refine the decoder training on only those trials that were initially identified as trials in which attention was oriented close to the target. All in all, the abovementioned studies confirm the association between the decoded readout of spatial attention and the observed task-related behavior of the subject. Despite the clarity of this relationship between the distance of the decoded attention to the real target (or distractor) position and the probability to respond to a target (or distractor), one intriguing question is why trials that are characterized by attention decoded at a similar distance from a target (or distractor) sometimes result in a correct detection (or a false alarm) and other times in a miss (or distractor rejection). Inter-neuronal correlations turn out to be significantly lower on the correct trials than on the miss or false alarm trials, suggesting that error trials might arise when the neuronal population is in a lower informational capacity state characterized by higher noise correlation values (Astrand et al., 2016; Ben Hadj Hassen and Ben Hamed, 2020), which will be explored in Section Noise correlation and neuronal population information capacity. Overall, this work thus demonstrates that, from one trial to the next, although attention is often assumed to be stable at the cued location, it is not quite often. Rather, attention explores space dynamically, shifting from one location to the next every 100 ms or so. Because the attentional dynamics is revealed through the decoding of attention-related information from population neuronal activity, this indicates that the attentional dynamics is subserved by rapid changes in the spiking rates of individual neurons, during the attention orienting delay, as shown in Figure 3C. Thus, while stable attention was generally assumed to be subserved by persistent neuronal responses, this section demonstrates that attention is dynamic and is subserved by dynamic neuronal responses and not by persistent neuronal activity.

### Attention Explores the Space Rhythmically

Classically, the spotlight theory of attention assumes that attention is only focused at one location of space at a time [Eriksen and St. James, 1986; see the discussion in Posner and Petersen (1990), Gaillard and Ben Hamed (2020)]. This view posits that it is possible to shift the spotlight of attention from one location to another, independent of the eye position and adjustment of its size to the attended location like a zoom lens. Thus, it intrinsically assumes a certain degree of flexibility of attention. Recent behavioral evidence (Venables, 1960; Landau and Fries, 2012; Dugue and VanRullen, 2014; Song et al., 2014) shows that, instead of a smooth and continuous behavior, spatial attention samples the visual environment rhythmically, leading to fluctuating periods of perceptual sensitivity [see VanRullen (2016) for a review]. In other words, these studies suggest that attention and perception might not be attached to a specific location in space (e.g., the cued location), but rather exhibit a temporal rhythmicity between relevant spatial locations [but see Brookshire (2021) for a critical perspective on these observations].

In agreement with these behavioral studies, neurophysiological evidence indicates that the brain activity underlying visual attention, as measured from the local field potentials (LFPs), is rhythmic in the theta band (4–8 Hz) (Lakatos et al., 2008; VanRullen, 2013, 2016; Fiebelkorn et al., 2018; Spyropoulos et al., 2018). For example, Fiebelkorn et al. (2018) show that, in the execution of a cued detection task, monkeys' ability to detect a target fluctuates rhythmically as a function of the time from cue onset, at a rhythm of 4 Hz. Importantly, the likelihood to correctly respond to the target is predicted by the phase of the ongoing oscillations in the prefrontal LFPs in the same frequency band with respect to the cue onset.

Fluctuations in the behavioral attentional performance and in the prefrontal LFP power are in sharp contrast with the notion of a stable prefrontal attentional code following a cue orientation (Astrand et al., 2015). In order to directly address this question, Gaillard et al. (2020) extend the work by Astrand et al. (2016) to a temporally highly resolved decoding of spatial attention (over 50 ms neuronal recording windows, instead of 150 ms). At this temporal resolution, rhythmic fluctuations in the prefrontal attentional information are observed in the 7–12 Hz alpha range. As described in Figure 3, these attentional oscillations are not observed at an individual cell level; however, they become apparent when averaging on multiple simultaneously recorded signals (Figure 3C). The rhythmic fluctuations in the prefrontal attentional information are decoded as spatio-temporal (*x, y*) attentional traces, and systematic changes in the location of the decoded attentional spotlight (or attentional saccade) can be seen at a frequency of ~8 Hz. These traces clearly show that, during the cue-to-target interval, attention explores both the cued locations but also uncued spatial locations. Importantly, this spatial exploration of space by attention also exists prior to attentional cueing, suggesting that the rhythmicity of attention is a default mode. In addition, how the prefrontal attentional trace explores the space varying from one behavioral task to another, indicating that it is under a top-down control. Overall, Gaillard et al. (2020) propose that the rhythmic variations in the attentional exploration subtend an efficient compromise between the exploitation of the prior information and the exploration of the novel information within a given trial.

All these abovementioned studies work under the assumption that the attentional spotlight is unique, a paradigm that has been driving most neurophysiological studies (Moran and Desimone, 1985; Niebur and Koch, 1994; Lee et al., 1999; Martínez et al., 1999; Reynolds et al., 2000; Corchs, 2002). However, this model of attention is limited when one needs to attend to more than one object at a time. In this context, other models of attention have been proposed. One of them is the zoom lens hypothesis, which considers a single attentional spotlight that is able to select the information from multiple locations by adjusting its size (Eriksen and Yeh, 1985; Eriksen and St. James, 1986). Another model proposes that the spotlight can be split, and attention may be simultaneously deployed to multiple spatial regions (Awh and Pashler, 2000; McMains and Somers, 2004; Niebergall et al., 2011; Mayo and Maunsell, 2016). Of utmost interest and relevance, current neurophysiological paradigms do not allow a direct evaluation of these concurrent theoretical models of attention.

All this taken together support the idea that seemingly persistent prefrontal single neuronal and population activity is actually highly dynamic, reflecting complex ongoing endogenous (i.e., covert) processes. These dynamic processes can only be accessed at the single-trial level because they (and their specific associated informational content) vary from one trial to the next. When averaged, these trial-to-trial variations are wiped out.

## State Dependence of Prefrontal Neuronal Activity

In the previous section, we address the sources of neuronal response variability that correlate with the dynamic nature of attention at the single-cell level and at the population level. In contrast, in this section, we consider the neurophysiological markers that impact the neuronal population information capacity and overt behavior irrespective of individual neuronal spiking rates and also irrespective of whether neuronal activity is persistent or not. We first discuss the noise correlation across the neuronal population and how it impacts the neuronal population informational capacity. Then, we discuss global fluctuations in the neuronal population attentional information that occur irrespective of ongoing attentional processes but directly impact both attentional neuronal responses and behavior.

### Noise Correlation and Neuronal Population Information Capacity

Noise correlations have been shown to critically impact both cortical signal processing and behavioral performance in different domains such as learning and attention (Ben Hadj Hassen and Ben Hamed, 2020). Shared neuronal variability across all recorded neurons is independent of the shared neuronal variability induced by the signal (Ben Hadj Hassen et al., 2019). The accuracy of a population code depends on not only the neuronal correlation arising from a common input (such as sensory information or cognitive control information) but also on the neural correlations that arise from a stimulus-independent activity. Indeed, a noise correlation is shown to interfere with the informational capacity of neuronal populations to represent a given variable and the resilience of this neuronal population to noise interference [see Averbeck et al. (2006), for a review]. For example, Froudarakis et al. (2014) show that the less correlated the firing pattern in V1 neurons, the higher the discriminability of the population code between the different visual stimuli. Likewise, lower noise correlations have also been associated with more efficient memory storage (Olshausen and Field, 2004). However, the relationship between the noise correlation and informational capacity is not straightforward. Indeed, it has been shown that inter-neuronal noise correlations can either improve the overall informational capacity, and hence decoding accuracy or, on the opposite, degrading decoding accuracy mostly depending on how the strength of noise correlations is compared to the strength of signal correlations (Averbeck et al., 2006; Moreno-Bote et al., 2014; Ben Hadj Hassen and Ben Hamed, 2020). How much decoding benefits from the decorrelated neuronal activities thus depends on a variety of experimental and neurophysiological factors (Ben Hadj Hassen and Ben Hamed, 2020).

Astrand et al. (2016) show that the noise correlation in prefrontal neuronal populations is predictive of the overall behavioral performance, which is lower on upcoming correct trials than on upcoming misses or false alarms. They further show that the fluctuations in noise correlations are very slow as noise correlations are globally lower on a given trial either on correct or error trials when the previous trial was a correct trial. In contrast, noise correlations are globally higher in a given correct of error trial when the previous trial was a miss trial. This strongly indicates that more global mechanisms are mediated among other things by a noise correlation, interact with spatial attention processes, and significantly contribute to overt behavioral performance. Importantly, the slow fluctuations in noise correlations are independent of variations in the overall spiking level, confirming that they reflect the state of connectivity of a given neuronal population.

Fluctuations in a noise correlation (and thus in the overall population informational capacity) are also observed at slower timescales than at a trial level. Indeed, Ben Hadj Hassen et al. (2019) show that noise correlations are lower on difficult tasks as compared to easy tasks. This suggests that an active mechanism might contribute to adjusting the neuronal noise correlation to ongoing behavioral demand, thus high-noise correlation states corresponding to a default “relaxed” population state.

In addition, fluctuations in a noise correlation are also characterized at faster timescales. For example, Ben Hadj Hassen et al. (2019) show that prefrontal noise correlations fluctuate within two distinct frequency bands, a high alpha frequency range (10–16 Hz) and a beta frequency band (20–30 Hz). These fluctuations that are independent of fluctuations in neuronal spiking rates are shown to impact behavioral performance and are reproduced in three different behavioral tasks. The authors propose that selective changes of frequency in spike-LFP phase coherence might account for these fluctuations in a noise correlation. Likewise, Womelsdorf et al. (2012) show the fluctuations in V1 noise correlation at an even higher gamma frequency (60–80 Hz), correlating both with the changes in performance and with orientation selectivity as a function of the phase in the gamma cycle.

Overall, the results suggest that noise correlations vary at different timescales, from a very slow to fast, suggesting fluctuations in the overall neuronal population capacity in the same timescale. This is actually confirmed by the observation that the variations in behavioral performance correlate with the variations in noise correlation. The studies cumulatively indicate that the information capacity in a given neuronal population is not only determined by spiking patterns, as described in the previous section but also by inter-neuronal noise correlations, a neurophysiological metric, which is decoupled from the firing rates and still anticorrelated with the attentional information and fluctuates in time in multiple scales. Thus, this further weakens the link between attentional processes and persistent activity.

However, other statistical features of the neural population are also reported to impact the amount of encoded information, such as changes in the network state, neuronal tuning, and global activity modulations (Cohen and Newsome, 2008; Harris and Thiele, 2011; Gutnisky et al., 2017; Verhoef and Maunsell, 2017). In addition, there is no consensus on whether the statistical features of population responses that affect the amount of information encoded in the neural populations also impact behavior (Arandia-Romero et al., 2017; Panzeri et al., 2017). In a very recent paper, Nogueira et al. (2020) have investigated which features of neural population responses most determine the overall amount of encoded information and behavioral performance. Examining neurons in two different brain areas (the middle temporal area and the lateral PFC), they found that the amount of information encoded in a population and behavioral performance was highly determined by the two statistical features: (1) the length of the vector joining the mean population responses in different experimental conditions [population signal (PS), corresponding to the distance, in lower-dimensional space, between the neuronal response patterns in different conditions] and (2) the inverse population co-variability projected onto the direction of the PS vector [projected prevision (PP), corresponding to the degree of alignment between the low-dimensional representation of the neuronal responses of each experimental condition]. Importantly, keeping the two parameters fixed, the authors did not find a clear relationship between the noise correlation and the amount of encoded information; however, they found a covariation between the latter parameter with PP and PS that could explain the observed effects of noise correlation in the amount of encoded information in the prior studies.

### Very Slow Fluctuations in Prefrontal Information Capacity

Until now, we have shown that prefrontal activity during the processing of the attention information is highly dynamic, showing rhythmic fluctuations in the attentional information in the alpha range (~10 Hz). The fluctuations are associated with a behavioral outcome of the subject, shedding new light on how the attentional system holds the information in a short timescale. However, little is known about the dynamics of the attention information in longer timescales (in the range of minutes and even hours). In this context, previous studies have shown that when attention is actively sustained in time, such as in the context of long-lasting cognitive demands, and the performance seems to decrease (Proctor et al., 1996; Lockley et al., 2004; Bonnefond et al., 2010; Virtanen and Kivimäki, 2018). A recent work by Gaillard et al. (2021) suggests that this might not always be the case. Indeed, they report that behavioral performance in a visual attentional task fluctuates by up to 10% at an ultra-slow rhythm of 4–7 cycles per hour (every 9–15 min), coinciding with phase-locked rhythmic fluctuations in the accuracy of visual and spatial attention information in the PFC. The behavioral and neuronal information fluctuations were not associated with concurrent variations in the spiking rate. However, an enhanced theta (~6 Hz) and beta (~24 Hz) oscillatory activity in LFP and an enhanced alpha (~10 Hz) in LFP coherence were observed during high behavioral performance epochs. Overall, this thus adds a level of complexity to prefrontal activity, in particular during cognitive processing (spatial attention delays), as prefrontal attentional population coding appears to be impacted by long-range distal signals (possibly related to states of vigilance and/or of fatigue and energy depletion), shifting from a high processing efficiency state (associated with enhanced visual and attentional coding accuracies), and a low processing efficiency state (associated with degraded visual and attentional coding accuracies).

## Prefrontal Neuronal Population Activity Reflects Multiple Processes

### Prefrontal Cortical Population Activity and Mixed Selectivity

We have already described the different population activity regimes that were region-specific but also dependent on the source of encoding information (e.g., position or color of the cue, Section 2.2). Prior studies have demonstrated a specific property of PFC neurons (specifically, the neurons from area 46 in the lateral PFC) called mixed selectivity (Rigotti et al., 2013; Parthasarathy et al., 2017). This property, which has also been reported in the FEF (Brincat et al., 2018; Khanna et al., 2020), allows that the neurons exhibit complex patterns of responses reflecting simultaneously different task-related parameters. Due to the complex functional pattern of activation, single-neuron recording studies on the PFC have found difficulties in relating the parameters to a specific neural activity, since the neurons will encode multiple parameters simultaneously, and the given spiking rate cannot unambiguously be assigned to the specific state of a given function. Approaches based on the average activity from the pre-selected neurons based on the specific criteria across multiple trials have been extensively used as a state-of-the-art in multiple neurophysiological studies (e.g., Ibos et al., 2013). However, these approaches, even though useful in identifying some specific information processes, elude most of the structure of the single-cell responses (Wohrer et al., 2013). This is because complex patterns of behavior might rely on the coordination of different neural mechanisms at a population level rather than on the activity of single neurons. In this context, the analysis of the neural population as a whole allows the extraction of features in the data using dimensionality reduction methods [see Cunningham and Yu (2014), for a review]. One of the methods is the principal component analysis (PCA), which consists of extracting an ordered set of orthogonal directions capturing the greatest variance in data. An important caveat of this method is that the obtained low-dimensional space captures all types of variances—without unmixing the underlying sources. Therefore, mixed selectivity remains in the data after the reduction of dimensionality, preventing from associating a task- or even behavior-specific variance to individual components. This issue has recently been solved by addressing dimensionality reduction methods with explicit information about the variance related to the parameters (Machens, 2010; Mante et al., 2013; Kobak et al., 2016). Specifically, demixed PCA (dPCA; Machens, 2010) is a dimensionality reduction method that aims to decompose the data into features easily interpretable with respect to specific parameters while preserving the original data as much as possible (Kobak et al., 2016).

### Unmixing Spatial Attention and States of Inattention (or Attentional Lapses) From Prefrontal Population Activity

One open question in the attention research is to what extent the readout of the attention information fully accounts for the reported behavior of the subject. Previously, it has been shown that the position of attention with respect to the actual position of the stimulus to be processed accounted for behavior, such that the closer the decoded attentional spotlight to the stimulus (or distractor) prior to the stimulus onset, the more likely the behavioral response to this stimulus (or distractor) (Astrand et al., 2016, 2020). However, these studies show that in trials with a similar distance between the decoded position of attention and the actual cued position, the behavioral outcome could be different, the subject sometimes producing a correct response, and other times producing an error response. This suggests that, on top of the attentional dynamics, different neural states of activity might influence how the system is able to exploit the attention information. In a very recent study, Amengual et al. (2021) isolate, from the PFC population activity, components specifically associated either to the position of the decoded attentional spotlight relative to the expected target position or to the behavioral outcome (hit vs. miss) using dPCA. They consistently find that the components encoded the specific information from each parameter, respectively (attention and reported behavior). Interestingly, they find that the information about the two components partially overlapped (they are not orthogonal), the smaller the overlap the higher the behavioral gain associated with an efficient attention orientation. In other words, the smaller the overlap, the lower the interference at the behavioral level between the spatial attention orientation and the state of inattention encoded by the prefrontal neuronal population.

The results shed new light on the extent to which the system is able to use this information to optimize behavior. It suggests that an accurate performance involving an active engagement in an attentional task depends not only on the active attentional control and readout of the attended information but also on its integration with the activity associated with more general neural states that might correspond to levels of distractibility or impulsivity that allow access to the attended information. In addition, the results call for a functional reconsideration of persistent activity. Indeed, the multiplexing of the multiple states or features in a single population results in an apparent sustained activity. However, the precise informational content of this persistent activity can only be accessed by splitting it into well-defined functional components.

## Conclusion and Perspectives

Electrophysiological studies employed for recording individual cells of the primate PFC have shown clear evidence of persistent spiking activity for visual delay tasks associated with different aspects of cognition. In the present work, we have reviewed the role of the so-called persistent activity in the domain of attention orienting during the delayed visual attention tasks. In this context, classical approaches in the field mostly based on the analysis of single-cell recordings in the FEF- and LIP-averaging neuronal activity across multiple trials have shown that the sustained neuronal spiking activity during the cue-to-target time interval depends on the spatial preference of the cell, being higher when attention is located in the preferred spatial position of the neuron from both areas. However, the “persistence” of the persistent activity has been repeatedly questioned [see Constantinidis et al. (2018), Lundqvist et al. (2018a)].

Accordingly, we have shown clear evidence that, at a single-trial level, the spiking activity of individual neurons is sparse and very heterogeneous across successive trials. In particular, we show that this applies to spatial attention, and that spatial attention is not attached to a specific cued location in space, but rather expresses intrinsic oscillatory dynamics covering the whole visual space in a rhythmic manner at approximately 8 Hz, impacting behavioral performance (Lakatos et al., 2008; Dugue and VanRullen, 2014; VanRullen, 2016; Fiebelkorn et al., 2018; Spyropoulos et al., 2018; Gaillard et al., 2020). In addition, we also show that the neuronal population codes for spatial attention vary at a very slow rhythm of a few cycles per hour. Although the impact of the oscillations on behavioral performance is very strong, their origin is still unknown. The fluctuations in the prefrontal spatial attention codes cannot be tracked on the single neuronal responses, and only become apparent when a larger neuronal population is considered. Lastly, using dimensionality reduction techniques, we consider an additional degree of complexity of delay-related prefrontal activity, identifying specific neuronal sources of variance associated with overt behavioral performance (correct vs. errors) and attention, respectively (Amengual et al., 2021). While most studies on mixed selectivity in the prefrontal neuronal population have focused on task-related information coding, here we consider a condition in which mixed selectivity is associated with a task-independent variable (a state of inattention or attentional lapse) that dynamically and transiently interferes with task-related processes.

Overall, we thus provide a systematic deconstruction of the idea of the persistence of the neuronal activity in the context of attention orienting, and we describe multiple sources of neuronal dynamic processes in the “silent” epochs of cognitive tasks in multiple time scales. An important challenge that remains to be addressed is how this dynamic is organized both at the mesoscopic level of the cortical area and its layers and at the level of the functional network.

## Author Contributions

SB and JA: conceptualization, figures, writing—original draft, and writing–review and editing. SB: funding acquisition and supervision. All authors contributed to the article and approved the submitted version

## Conflict of Interest

The authors declare that the research was conducted in the absence of any commercial or financial relationships that could be construed as a potential conflict of interest.

## Supplementary Material

Supplementary Figure 1 Description of the rapid serial visual presentation (RSVP) task (Ibos et al., 2013). Monkeys have to fixate a central point while a first stream of visual stimuli is presented (stimuli changing every 200 ms). After a few stimuli, a second stream of visual stimuli is presented contralateral to the first stream. A cue is then presented in the first visual stream. The cue can either be green instructing the monkey to maintain attention on this first visual stream (stay cue) because the target will be presented in this stream. Alternatively, the cue can be red, instructing the monkey to shift attention to the second visual stream (shift cue) because the target will be presented in this stream. The cue can thus be red or green (color dimension), presented in the left or in the right visual streams (position dimension), or instruct attention to be oriented to the left or to the right visual streams (attention dimension). Monkeys are rewarded to maintain fixation all throughout the trial and respond to the target presentation with a manual response as fast as possible.

Supplementary Figure 2 Schema of the construction of a classifier for two-dimensional (2D; *x,y*) readout of the attention information. **(A)** Schema of an electrode with two contacts recording multiunit activity (MUA) activity obtained from two different neurons. Each of the two neurons is tuned to two classes of information (different positions), represented by red and blue colors. **(B)** To quantify the amount of information in the data, a regularized linear regression is applied. *R*: mean neural response in a specific sliding window [shaded gray in **(A)**], W is the synaptic weights representing the contribution of each cell to the final readout and C is a 2D vector describing the two possible classes. A Tikhonov-regularized version is used in order to minimize the cost function to avoid overfitting. A linear decoder estimates a decision boundary of the classifier when discriminating between the population response to class 1 or to class 2 events. **(C)** Each output of the decoder represents an (*x,y*) position relative to the decision boundary, representing the decoded position of the attentional locus in the visual space. **(D)** Decoding procedure applied along the whole cue-to-target interval provides time-resolved decoding of the attentional trajectory associated with each class (position).
