# Supplementary figures and images for: Revisiting Persistent Neuronal Activity During Covert Spatial Attention
Source: Front Neural Circuits. 2021 Jun 30;15:679796. doi: 10.3389/fncir.2021.679796 (PMC8278237; doi:10.3389/fncir.2021.679796)

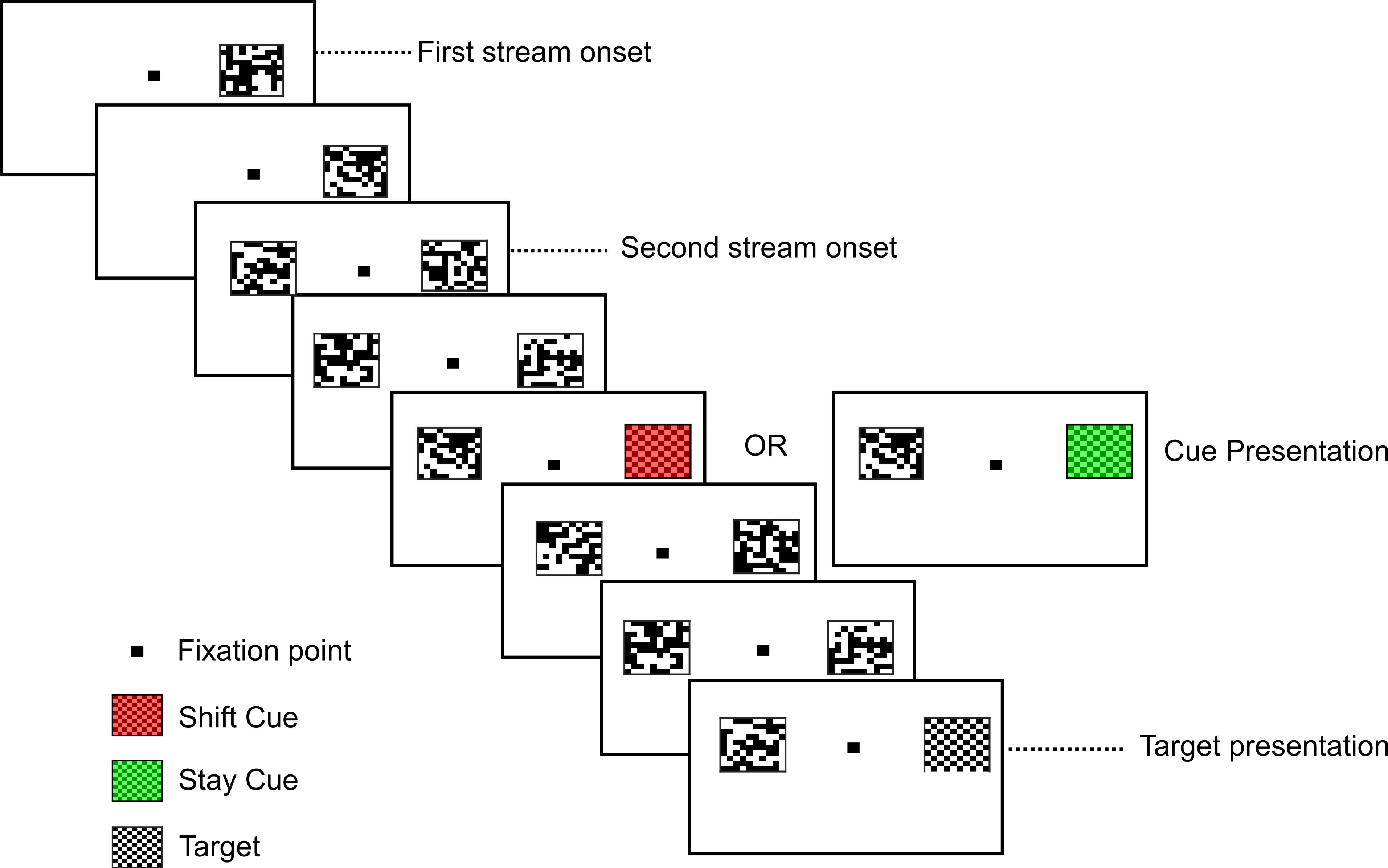

Supplement: Supplementary Figure 1 — Description of the rapid serial visual presentation (RSVP) task (Ibos et al., 2013). Monkeys have to fixate a central point while a first stream of visual stimuli is presented (stimuli changing every 200 ms). After a few stimuli, a second stream of visual stimuli is presented contralateral to the first stream. A cue is then presented in the first visual stream. The cue can either be green instructing the monkey to maintain attention on this first visual stream (stay cue) because the target will be presented in this stream. Alternatively, the cue can be red, instructing the monkey to shift attention to the second visual stream (shift cue) because the target will be presented in this stream. The cue can thus be red or green (color dimension), presented in the left or in the right visual streams (position dimension), or instruct attention to be oriented to the left or to the right visual streams (attention dimension). Monkeys are rewarded to maintain fixation all throughout the trial and respond to the target presentation with a manual response as fast as possible. [file Image_1.TIF]

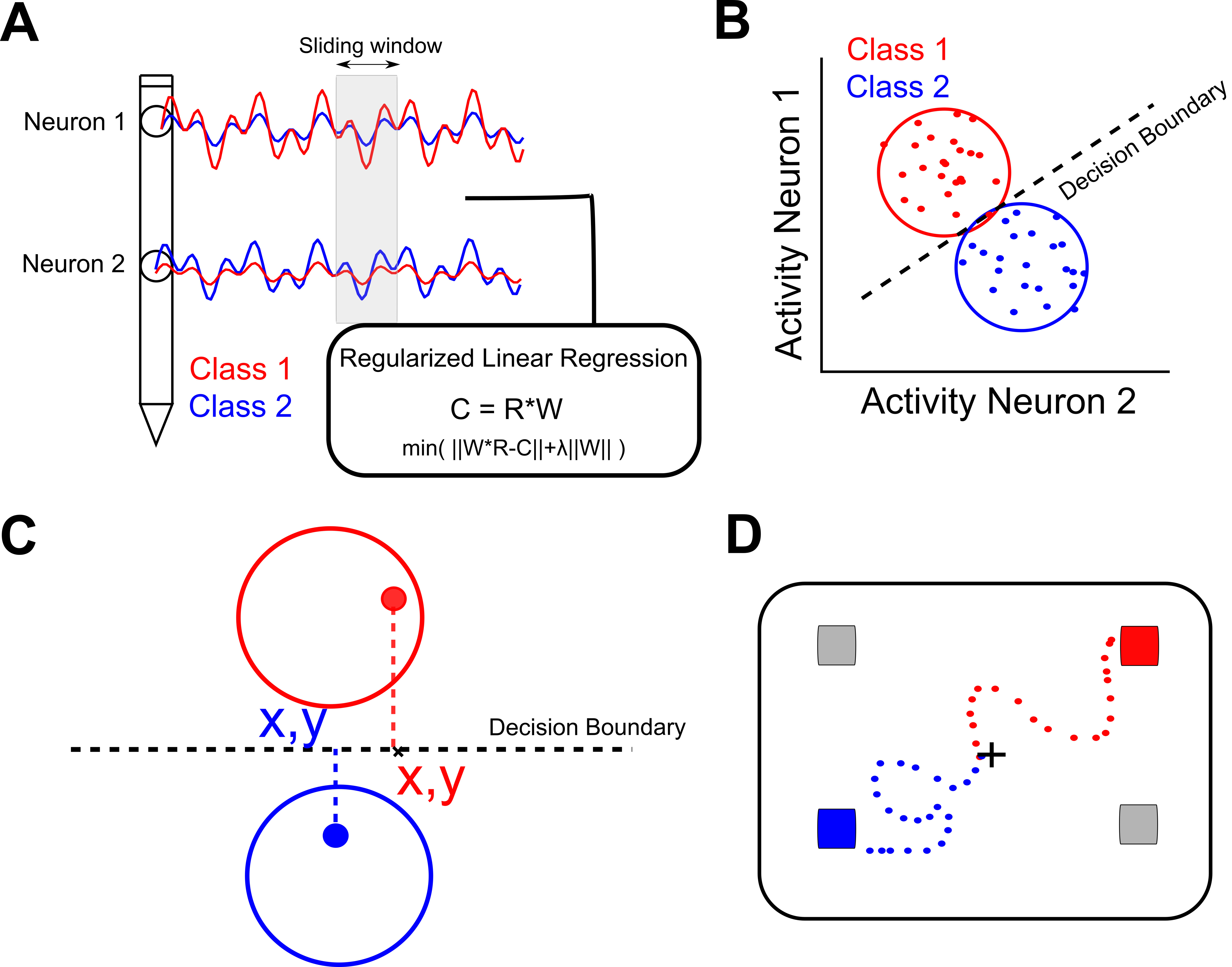

Supplement: Supplementary Figure 2 — Schema of the construction of a classifier for two-dimensional (2D; x,y) readout of the attention information. (A) Schema of an electrode with two contacts recording multiunit activity (MUA) activity obtained from two different neurons. Each of the two neurons is tuned to two classes of information (different positions), represented by red and blue colors. (B) To quantify the amount of information in the data, a regularized linear regression is applied. R: mean neural response in a specific sliding window [shaded gray in (A)], W is the synaptic weights representing the contribution of each cell to the final readout and C is a 2D vector describing the two possible classes. A Tikhonov-regularized version is used in order to minimize the cost function to avoid overfitting. A linear decoder estimates a decision boundary of the classifier when discriminating between the population response to class 1 or to class 2 events. (C) Each output of the decoder represents an (x,y) position relative to the decision boundary, representing the decoded position of the attentional locus in the visual space. (D) Decoding procedure applied along the whole cue-to-target interval provides time-resolved decoding of the attentional trajectory associated with each class (position). [file Image_2.TIF]
